# Structure of alumina glass

**DOI:** 10.1038/s41598-021-04455-6

**Published:** 2022-01-11

**Authors:** Hideki Hashimoto, Yohei Onodera, Shuta Tahara, Shinji Kohara, Koji Yazawa, Hiroyo Segawa, Motohiko Murakami, Koji Ohara

**Affiliations:** 1 Department of Applied Chemistry, School of Advanced Engineering, Kogakuin University, 2665-1 Nakano, Hachioji, Tokyo 192-0015 Japan; 2 Institute for Integrated Radiation and Nuclear Science, Kyoto University, 2-1010 Asashiro-nishi, Kumatori-cho, Sennan-gun, Osaka 590-0494 Japan; 3 Research Center for Advanced Measurement and Characterization, National Institute for Materials Science, 1-2-1 Sengen, Tsukuba, Ibaraki 305-0047 Japan; 4 Department of Physics and Earth Sciences, Faculty of Science, University of the Ryukyus, 1 Chihara, Nakahara cho, Nakagami-gun, Okinawa, 903-0213 Japan; 5 Department of Earth Science, ETH Zürich, Clausiusstrasse 25, 8092 Zürich, Switzerland; 6 JEOL RESONANCE Inc., 3-1-2 Musashino, Akishima, Tokyo 196-8558 Japan; 7 Research Center for Functional Materials, National Institute for Materials Science, 1-1 Namiki, Tsukuba, Ibaraki 305-0044 Japan; 8 Diffraction and Scattering Division, Japan Synchrotron Radiation Research Institute, 1-1-1 Kouto, Sayo-gun, Hyogo, 679-5198 Japan

**Keywords:** Structure of solids and liquids, Characterization and analytical techniques

## Abstract

The fabrication of novel oxide glass is a challenging topic in glass science. Alumina (Al_2_O_3_) glass cannot be fabricated by a conventional melt–quenching method, since Al_2_O_3_ is not a glass former. We found that amorphous Al_2_O_3_ synthesized by the electrochemical anodization of aluminum metal shows a glass transition. The neutron diffraction pattern of the glass exhibits an extremely sharp diffraction peak owing to the significantly dense packing of oxygen atoms. Structural modeling based on X-ray/neutron diffraction and NMR data suggests that the average Al–O coordination number is 4.66 and confirms the formation of OAl_3_ triclusters associated with the large contribution of edge-sharing Al–O polyhedra. The formation of edge-sharing AlO_5_ and AlO_6_ polyhedra is completely outside of the corner-sharing tetrahedra motif in Zachariasen’s conventional glass formation concept. We show that the electrochemical anodization method leads to a new path for fabricating novel single-component oxide glasses.

## Introduction

Oxide glasses, e.g., window glass, fiber glass, optical glass, and the cover glass of a smart phone are indispensable materials in our daily life. However, the fabrication of a novel single-component oxide glass is challenging particularly when a conventional melt–quenching method is used because the glass forming ability is governed by the viscosity of a high-temperature melt. Indeed, Angell proposed the concept of “fragility” to understand the relationship between the viscosity and the glass forming ability^1^. The basic idea behind the formation of covalent glass is the corner-sharing tetrahedral motif proposed by Zachariasen in 1932^2^. Sun classified single-component oxides into glass formers, glass modifiers, and intermediates^3^. SiO_2_, B_2_O_3_, P_2_O_5_, and As_2_O_3_ are typical glass formers, in which the cation–oxygen coordination number is 3 or 4 and the glass network is formed by corner-sharing oxygen atoms. Alkali and alkali earth oxides are typical glass modifiers; they cannot form glass, but they can modify the network formed by a network former by breaking cation–oxygen bonds in the network and/or occupy voids^4,5^. Alumina (Al_2_O_3_) can be considered as an intermediate, because it can be both a glass former and a glass modifier in binary oxide glasses, although Al_2_O_3_ cannot solely form glass.

Al_2_O_3_ has many applications, e.g., in cements, substrates of electronic materials, and high-temperature crucibles. As mentioned above, it is impossible to prepare Al_2_O_3_ glass by the melt–quenching method; hence, sol–gel methods were used to prepare amorphous samples for studying optical properties^6,7^ and behaviors at high temperatures^8^. Another approach is the fabrication of thin films, such as the highly ductile amorphous Al_2_O_3_ thin films that have recently been reported^9^. However, the structure of amorphous Al_2_O_3_ is still largely unknown owing to a very limited number of structural studies. Lamparter and Kniep reported the formation of AlO_4_ tetrahedra with corner-sharing oxygen atoms as confirmed by neutron and X-ray diffraction measurements with the aid of the reverse Monte Carlo (RMC)^10^ modeling technique^11^. Hashimoto et al. reported the average Al–O coordination number of 4.7 determined by ^27^Al nuclear magnetic resonance (NMR) spectroscopic measurements^12^, whereas Lee and Ryu confirmed the formation of OAl_3_ triclusters by ^17^O NMR measurements^13^. Shi et al. have recently reported the comparison between amorphous Al_2_O_3_ and liquid Al_2_O_3_, and they concluded on the basis of molecular dynamics (MD) and empirical potential structure refinement (EPSR)^14^ simulations based on diffraction data^15^ that the Al–O coordination number is increased in amorphous Al_2_O_3_.

In this study, we have found that amorphous Al_2_O_3_ synthesized by the anodization of aluminium metal shows a glass transition by differential thermal analysis (DTA). We have performed ^27^Al solid-state NMR and high-energy X-ray and neutron diffraction measurements. Moreover, we constructed a structural model for the glass by a combined MD–RMC modeling technique to understand the structure of single-component *intermediate* oxide glass, because it is expected that the glass structure is inconsistent with Zachariasen’s rules.

## Methods

### Preparation of alumina glass

The Al_2_O_3_ sample was prepared according to our previous report^12^. High-purity (99.99%) aluminium sheets were immersed in 1.25 mol dm^−3^ NaOH for 20 s at 60 °C, washed with tap water, immersed in 3.9 mol dm^−3^ HNO_3_ for 60 s, and finally washed with deionized water to remove surface native oxides and contaminants. Constant-voltage anodization was performed for 80 V in 0.3 mol dm^−3^ H_2_CrO_4_ electrolyte for 2 h at 40 °C. After anodization, the sample was carefully washed with deionized water to remove residual electrolyte. A stepwise voltage reduction from formation voltage to ~ 60 V was performed in the same electrolyte to reduce barrier layer thickness. To detach the alumina from the aluminium substrates, anodic polarization was applied in a mixed solution of 1:4 vol% of perchloric acid (60%) and ethanol (99.5%) for 1 min at ~ 70 V. The detached sample was carefully washed with deionized water, dried at room temperature, and crushed into powder by an agate mortar. To remove physisorbed water, the powder sample was heat-treated at 300 °C for 4 h at a heating rate of 5 °C min^−1^ and were subsequently cooled in the furnace, as previously reported.

### Density measurement

The density measurement was performed on a helium pycnometer (AccuPyc 1340TC, Shimadzu-Micromeritics). Before the measurement, the sample was dried for 24 h at room temperature in vacuum.

### DTA measurement

The differential thermal analysis (DTA) experiment was performed on a Rigaku Thermo plus EVO apparatus. The sample was dried at a heating rate of 10 °C min^−1^ to 300 °C for 4 h and cooled in the furnace. After reaching 50 °C, the sample was heat-treated at a heating rate of 10 °C min^−1^ to 1350 °C.

### NMR measurements

The ^27^Al magic angle spinning (MAS) nuclear magnetic resonance (NMR) experiment was performed on a JEOL JNM-ECA800 (18.79 T) spectrometer at a ^27^Al Larmor frequency of 208.58 MHz. The sample was packed in zirconia rotors and spun at 20 kHz using a 3.2 mm HXMAS probe. The ^27^Al chemical shift δ_iso_ in parts per million (ppm) was referenced to an external 1 mol dm^−3^ AlCl_3_ solution (− 0.1 ppm). The ^27^Al single-pulse MAS spectrum was obtained using 6/π pulses (0.67 us) with a recycle delay of 1 s and 512 scans. The spectrum was decomposed into three components, and fitting parameters, namely, the average isotropic chemical shift ($\overline{{{\varvec{\updelta}}}_{\mathbf{i}\mathbf{s}\mathbf{o}}}$), the width of the Gaussian distribution of δ_iso_ (ΔCS), and the average quadrupolar coupling constant ($\overline{{\mathbf{C}}_{\mathbf{Q}}}$), were determined using the “Dmfit” program^16^ applying a simple Czjzek model. The errors of fitting values for δ_iso_ and other parameters were < 0.04% and < 0.3%, respectively. The average *N*_Al–O_ was determined using the following equation: $\overline{{{\varvec{N}}}_{{\varvec{A}}{\varvec{l}}-\mathbf{O}}}=\sum {\varvec{N}}{{\varvec{A}}}_{{\varvec{N}}}$, where *N* and *A*_*N*_ represent the number of oxygen atoms around a given aluminium atom and the relative ratio of the corresponding peak area, respectively.

### Diffraction measurements

The high-energy X-ray diffraction experiment was performed at the BL04B2 beamline at the SPring-8 synchrotron radiation facility, using a two-axis diffractometer dedicated to the study of disordered materials^17^. The energy of the incident X-rays was 61.4 keV. The raw data were corrected for polarization, absorption, and the background, and the contribution of Compton scattering was subtracted by using standard data analysis software^17^. The neutron diffraction measurement was conducted on a high intensity total diffractometer, NOVA^18^, installed at BL21 of the Materials and Life Science Experimental Facility at the J-PARC spallation neutron source. The wavelength range of the incident neutron beam was 0.12 Å < *λ* < 8.3 Å. The glass sample was transferred into vanadium-nickel null alloy cell 6 mm in diameter. The observed scattering intensity for the sample was corrected for instrumental background, attenuation of the sample and cell, and then normalized by the incident beam profile. All corrected data were normalized to give a Faber–Ziman^19^ total structure factor *S*(*Q*). A Lorch^20^ modification function was used in Fourier transform.

### Structure modelling

We combined MD simulation with NVT ensemble–reverse Monte Carlo (RMC) modelling for structure modelling. The MD simulation was performed using the LAMMPS package^21^ and RMC modellings were performed using the RMC ++code^22^.

In the case of *l*-Al_2_O_3_, we used the Born–Mayer-type pair potential in the MD simulation given as

1 $${\varvec{\phi}}\left({{\varvec{r}}}_{{\varvec{i}}{\varvec{j}}}\right)={{\varvec{e}}}^{2}\frac{{{\varvec{q}}}_{{\varvec{i}}}{{\varvec{q}}}_{{\varvec{j}}}}{{{\varvec{r}}}_{{\varvec{i}}{\varvec{j}}}}+{{\varvec{B}}}_{{\varvec{i}}{\varvec{j}}}{\text{exp}}\left(\frac{-{{\varvec{r}}}_{{\varvec{i}}{\varvec{j}}}}{{{\varvec{R}}}_{{\varvec{i}}{\varvec{j}}}}\right).$$

Here, *e* is the elementary charge and *B*_*ij*_ and *R*_*ij*_ are the parameters accounting for the repulsion of ionic cells. *q*_Al_ =  + 3 and *q*_O_ =  − 2 are the charges of Al^3+^ and O^2–^, respectively. The *B*_*ij*_ values of 2.3708 × 10^−16^ J (Al–O), 2.4031 × 10^−16^ J (O–O) and zero (Al–Al) and the *R*_*ij*_ values of 0.29 Å (Al–O and O–O) and zero (Al–Al) are found in Ref.^23^. A random configuration composed of 10,000 atoms was prepared with respect to the experimental density (0.08630 Å^−3^). This configuration was heated to 5000 K and treated above 50,000 steps. Subsequentry, the configuration was cooled to 2400 K at a cooling rate of 1.3 K/ps. Eventually, the system was equilibrated at 2400 K for 100,000 steps. The long-range Coulomb interactions were calculated with standard Ewald summation and the simulation used periodic boundary conditions. A time step of 1 fs was used in the Verlet algorithm.

For *g*-Al_2_O_3_, the starting configuration, which contain 10,000 particles (Al, 4000: O, 6000) for *g*-Al_2_O_3_ was created using hard-sphere Monte Carlo (HSMC) simulation. The atomic number density is 0.09007 Å^−3^. The *r*-spacing for the calculations of the partial pair-distribution functions was set to 0.075 Å. Two kinds of constraints were applied: the closest atom–atom approach and the coordination number. The first one can avoid unreasonable spikes in the partial pair-distribution functions. The second forces aluminium atoms to coordinate to averaged 4.6 oxygen atoms within a cut off distance of 2.50 Å. After the HSMC simulation, RMC simulation was conducted to reproduce the X-ray *S*(*Q*) and neutron *S*(*Q*) data. Following the RMC simulations, the atomic configuration was optimized by MD simulation. The MD simulation was performed using pairwise additive interatomic terms of the form

2 $${\varvec{V}}\left({{\varvec{r}}}_{{\varvec{i}}{\varvec{j}}}\right)={{\varvec{e}}}^{2}\frac{{{\varvec{q}}}_{{\varvec{i}}}{{\varvec{q}}}_{{\varvec{j}}}}{{{\varvec{r}}}_{{\varvec{i}}{\varvec{j}}}}-\frac{{{\varvec{C}}}_{{\varvec{i}}}{{\varvec{C}}}_{{\varvec{j}}}}{{{\varvec{r}}}_{{\varvec{i}}{\varvec{j}}}^{6}}+{\varvec{D}}\left({{\varvec{B}}}_{{\varvec{i}}}+{{\varvec{B}}}_{{\varvec{j}}}\right){\text{exp}}\left(\frac{{{\varvec{A}}}_{{\varvec{i}}}+{{\varvec{A}}}_{{\varvec{j}}}-{{\varvec{r}}}_{{\varvec{i}}{\varvec{j}}}}{{{\varvec{B}}}_{{\varvec{i}}}+{{\varvec{B}}}_{{\varvec{j}}}}\right),$$

where the terms represent Coulomb, van der Waals, and repulsion energy, respectively. Here, *r*_*ij*_ is the interatomic distance between atoms *i* and *j*, *D* is a standard force constant 4184 JÅ^−1^ mol^−1^, *q*_*i*_ is the effective charge on atom *i* (*q*_Al_ = 1.17, *q*_O_ = − 0.78). The repulsive radius *A*_*i*_ values are 0.7852 Å (Al), 1.8215 Å (O); the softness parameter *B*_*i*_ values are 0.034 Å (Al), 0.138 Å (O); and the van del Waals coefficient *C*_*i*_ values are 36.82 Å^3^kj^1/2^mol^−1/2^ (Al), 90.61 Å^3^kj^1/2^mol^−1/2^ (O). The parameters *A*_*i*_, *B*_*i*_, *C*_*i*_ can be found in Ref.^24^. The optimization of the atomic configuration was performed by minimizing the energy using the conjugate gradient method. We confirmed that these parameters are in better agreement with diffraction data; in particular, a very sharp principal peak (PP) in *S*^N^(*Q*) for the glass was very well reproduced, while the parameters reported in Ref.^23^ underestimated the PP.

After the MD simulations, both configurations were refined by additional RMC simulations while constraining the Al–O coordination number, and the partial pair-distribution functions, *g*_*ij*_(*r*), within the first coordination shell to avoid unfavorable artifacts.

As a reference, we constructed three-dimensional structure model of *g*-SiO_2_ by combined MD–RMC simulation. The MD simulation of SiO_2_ glass was performed employing Born–Mayer type pair potentials, where the values *q*_Si_ and *q*_O_ are + 2.4 and − 1.2; the *B*_*ij*_ values were 21.39 × 10^−16^ J (Si–O), 0.6246 × 10^−16^ J (O–O) or zero (Si–Si); the *R*_*ij*_ values were 0.174 Å (Si–O), 0.362 Å (O–O) or zero (Si–Si)^25^. As the initial atomic configuration, 9000 atoms (Si, 3000: O, 6000) were randomly distributed in a cubic cell with respect to the experimental number density (0.06615 Å^−3^). The simulation temperature was maintained at 4000 K for 20,000 time steps, then the temperature was reduced to 300 K over 200,000 time steps. Finally, the system was equilibrated at 300 K for 50,000 time steps. After the MD simulation, the obtained atomic configuration was refined by additional RMC simulation. In the RMC refinement, the MD results for the Si–O coordination number and the bond angle distribution for O–Si–O, and the partial pair-distribution functions within the first coordination distance were used as constraints. The cut off distance for the constraints for coordination of silicon and O–Si–O bond angle distribution was set to 1.90 Å.

### Topological analyses

The bond angle distribution *B*(*θ*) was calculated as the number of bonds between *θ* and *θ* + Δ*θ*, which is dependent on the solid angle ΔΩ ∝ sin*θ* subtended at that value of *θ*. Thus, each bond angle distribution was plotted as *B*(*θ*)/sin*θ* to compensate for the effect of ΔΩ. The primitive^26,27^ (Al–O)_*n*_ ring size distributions for the *g*- and *l*-Al_2_O_3_ were calculated using the R.I.N.G.S. code^28,29^. The void analysis was conducted employing the pyMolDyn code^30^ with a cutoff distance of *r*_c_ = 2.50 Å.

## Results and discussion

**Figure 1:**
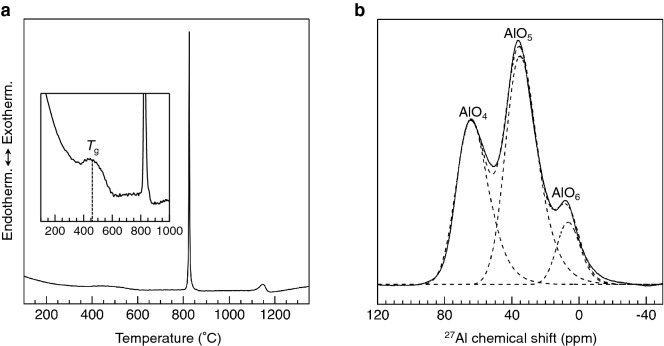
(**a**) DTA curve recorded at a heating rate of 10 °C min^−1^ for *g*-Al_2_O_3_. (**b**) ^27^Al solid state NMR spectrum for *g*-Al_2_O_3_.

Figure 1a shows a DTA curve for an Al_2_O_3_ sample. Sharp intense and broad weak exothermic peaks at ~ 830 and ~ 1150 °C, respectively, are observed. The former peak is assigned to the crystallization of γ-alumina from the amorphous phase and the latter peak is assigned to the phase transition from γ- to α-alumina^31^. Note that the slight baseline shift to the endothermic direction is observed in the low temperature region around 500 °C (inset of Fig. 1a), owing to glass transition, showing that the sample is Al_2_O_3_ glass (*g*-Al_2_O_3_). The starting point of the shift, i.e., the glass transition temperature, is determined to be ~ 470 °C. In general, common glass‐forming oxides have a ratio of glass transition temperature to melting point (*T*_g_/*T*_m_) of ~ 0.67^32^. On the other hand, the present *g*-Al_2_O_3_ shows a *T*_g_/*T*_m_ of ~ 0.32 (743 K/2345 K), which is extremely lower than that of general glass-forming oxides. The extraordinarily wide gap between *T*_g_ and *T*_m_ shows the low glass forming ability of alumina to maintain the deeply supercoiling state without the formation of crystal nucleus from the *T*_m_ = 2072 °C to *T*_g_ =  ~ 470 °C for realizing the glassy state.

Figure 1b shows a typical ^27^Al single-pulse MAS NMR spectrum normalized by the total peak area. This spectrum consists of three broad peaks located at around ~ 64, ~ 36, and ~ 7 ppm, which are assigned to four- (AlO_4_), five- (AlO_5_), and six-fold (AlO_6_) oxygen-coordinated polyhedra, respectively^12^. This spectrum is decomposed into the three components and the fitting result (dotted curve) is in good agreement with the measured data (solid curve). The fractions of AlO_4_, AlO_5_, and AlO_6_ are 37.5, 52.1, and 10.3%, respectively, and the average coordination number is determined to be 4.73. The values obtained here are slightly different from those of our previous report^12^; this variation depends on the resolution of the NMR equipment used. More precise values are obtained in the current study owing to the higher-resolution spectra obtained under higher magnetic fields. This precise local structural information is used as a constraint for the MD–RMC modeling as follows.

The mass density of *g*-Al_2_O_3_ is 3.05 g cm^−3^, which corresponds to the atomic number density of 0.0901 Å^−3^. This value is smaller than 4.00 g cm^-3^ of α-Al_2_O_3_ and slightly larger than 2.92 g cm^−3^ of *l*-Al_2_O_3_^33^. Note that the density of γ-Al_2_O_3_ is 3.59 g cm^−3^, which is between those of *g*-Al_2_O_3_ and α-Al_2_O_3_. This trend is very different from SiO_2_, in which the density of glass (2.20 g cm^−3^) is comparable to those of α-cristobalite (2.30 g cm^−3^) and β-cristobalite (2.20 g cm^−3^), implying that a large density difference between the glass and crystal in Al_2_O_3_ indicates a low glass forming ability in single-component oxide glasses.

**Figure 2:**
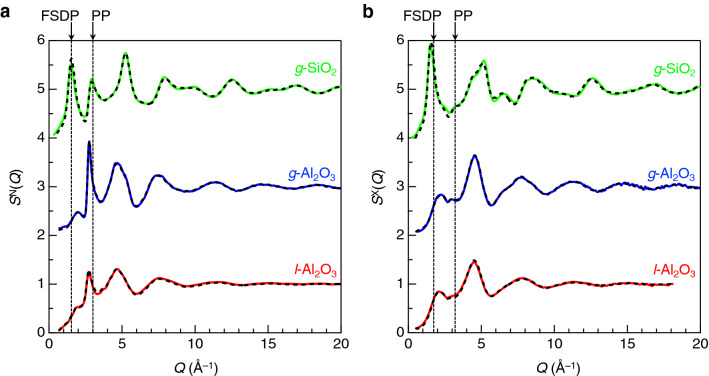
(**a**) Neutron total structure factors, *S*^N^(*Q*), together with the results of the MD–RMC simulations for *g*-Al_2_O_3_, *l*-Al_2_O_3_^33^, and *g*-SiO_2_^5^. (**b**) X-ray total structure factors, *S*^X^(*Q*), together with the results of the MD–RMC simulations for *g*-Al_2_O_3_, *l*-Al_2_O_3_^33^, and *g*-SiO_2_^5^. Coloured curve, experimental data; black broken curve, MD-RMC model. Successive curves are displaced upward by 2 for clarity.

Figure 2 shows neutron and X-ray total structure factors, *S*^N,X^(*Q*), for *g*-Al_2_O_3_ together with the results of MD–RMC simulation. For comparison, the results of silica glass (*g*-SiO_2_)^5^ and liquid alumina (*l*-Al_2_O_3_) at 2400 K^33^ are also shown. All the experimental *S*^N,X^(*Q*) data are well reproduced by the MD–RMC simulations. The first sharp diffraction peak (FSDP)^34^, which is from pseudo^35^ (quasi^36^) Bragg planes (successive small cages^37^) created along a void, is observed at *Q* = 1.52 Å^−1^ for *g*-SiO_2_, a typical glass forming oxide, whereas the FSDP observed at *Q* ~ 2 Å^−1^ is not prominent in *g*-Al_2_O_3_, suggesting the formation of a densely packed structure with a small void volume. In addition, *g*-Al_2_O_3_ shows an extraordinarily sharp PP^38^ in the neutron *S*(*Q*), but no PP is observed in the X-ray *S*(*Q*) owing to the small weighting factor of O–O correlations for X-rays, because PP reflects the packing of oxygen atoms^39^. Therefore, the extraordinarily sharp PP in the neutron *S*(*Q*), nearly twice sharper than those of *l*-Al_2_O_3_ and *g*-SiO_2_, suggests the extremely high packing fraction of oxygen atoms manifested by the high density of *g*-Al_2_O_3_. A similar behavior is found in the neutron diffraction data of *g*-SiO_2_ under a high pressure^40^. Both the overall profiles of *S*^N,X^(*Q*) for *l*-Al_2_O_3_ are broader than those for *g*-Al_2_O_3_, but the *S*^X^(*Q*) data of both *l*-Al_2_O_3_ and *g*-Al_2_O_3_ are more identical, suggesting that O–O correlations are different between *g*-Al_2_O_3_ and *l*-Al_2_O_3_.

**Figure 3:**
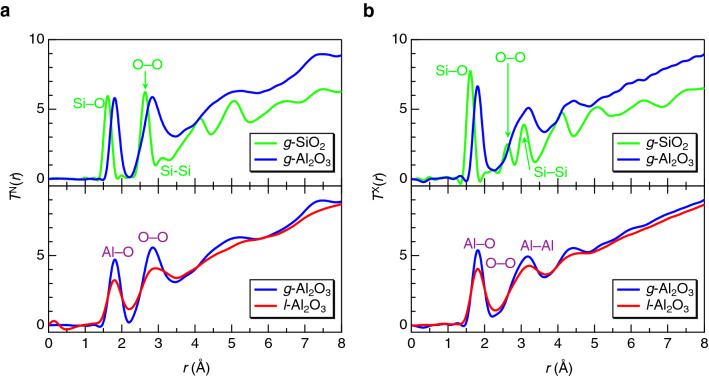
(**a**) Neutron total correlation functions, *T*^N^(*r*), for *g*-Al_2_O_3_, *l*-Al_2_O_3_^33^, and *g*-SiO_2_^5^. (**b**) X-ray total correlation functions, *T*^X^(*r*), or *g*-Al_2_O_3_, *l*-Al_2_O_3_^33^, and *g*-SiO_2_^5^. Upper and lower panel data were obtained by Fourier transform with *Q*_max_ = 25 and 18 Å^−1^, respectively. Note that the Al–Al correlation peak is not legibly marked owing to its small weighting factor for neutrons.

The neutron and X-ray total correlation functions, *T*^N,X^(*r*), for *g*-Al_2_O_3_, *l*-Al_2_O_3_^33^, and *g*-SiO_2_^5^ are shown in Fig. 3. The first peak observed at 1.81 Å in *T*^N,X^(*r*) for *g*-Al_2_O_3_ is assigned to the Al–O correlations. The second peak observed around 2.8 Å in *T*^N^(*r*) and that around 3.2 Å in *T*^X^(*r*) are assigned to the O–O and Al–Al correlations, respectively. Longer Al–O distances relative to *g*-SiO_2_ and the asymmetric Al–O correlation peak with a tail of ~ 2.4 Å indicate the formation of distorted AlO_*n*_ polyhedra with a coordination number higher than 4. The average Al–O coordination number calculated using the area of the first correlation peak of *T*^N^(*r*) is 4.6 ± 0.2, which is in agreement with the NMR result of 4.73 (37.5% of AlO_4_; 52.1% of AlO_5_; and 10.3% of AlO_6_) and larger than 4.4 in *l*-Al_2_O_3_. Such a larger coordination number, which is often observed in nonglass-forming high-temperature oxide melts^37,41^, cannot be observed in the typical glass-forming oxides. The overall profile for *g*-Al_2_O_3_ is similar to that for *l*-Al_2_O_3_, but the Al–O and O–O correlation peaks for *g*-Al_2_O_3_ are sharper than those for *l*-Al_2_O_3_, as apparently observed in *T*^N^(*r*). This behavior suggests that the packing of oxygen atoms in glass could differ from that in high-temperature liquid.

**Figure 4:**
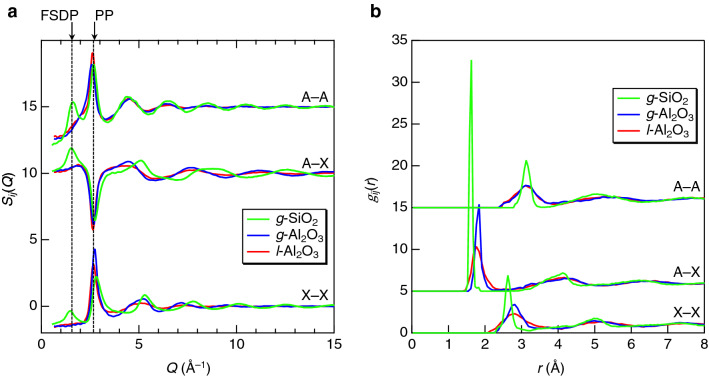
(**a**) Partial structure factors, *S*_*ij*_(*Q*), for *g*-Al_2_O_3_, *l*-Al_2_O_3_, and *g*-SiO_2_. (**b**) Partial pair distribution functions, *g*_*ij*_(*r*), for *g*-Al_2_O_3_, *l*-Al_2_O_3_, and *g*-SiO_2_. A = Si or Al and X = O.

Figure 4a shows the partial structure factors, *S*_*ij*_(*Q*), derived from the MD–RMC models for *g*-Al_2_O_3_ and *l*-Al_2_O_3_ together with those for *g*-SiO_2_. All the *S*_*ij*_(*Q*) give a positive peak at the FSDP position in *g*-SiO_2_, but there is no positive peak at the expected FSDP position in *g*-Al_2_O_3_. It is confirmed that the PP comprises the sum of positive correlations of A–A and X–X and negative correlations of A–X. As can be seen in Fig. 2b the PP is absent because the positive correlations of A–A and X–X are completely canceled by the A–X correlations in the *S*^X^(*Q*) of *g*-SiO_2_ and *g*- and *l*-Al_2_O_3_. On the other hand, the contribution of the X–X correlations at PP position is largely enhanced in the *S*^N^(*Q*) (see Fig. 2a) due to large weighting factors of O–O correlations for neutrons. The positive O–O PP for *g*-Al_2_O_3_ is sharper than that for *l*-Al_2_O_3_, resulting in the exceptionally sharp PP observed in the *S*^N^(*Q*) for *g*-Al_2_O_3_. Both the absence of the FSDP as mentioned above and the sharp PP in the *S*^N^(*Q*) mainly originated from the O–O correlations allow us to expect the formation of the dense oxygen packing in *g*-Al_2_O_3_. The partial pair distribution functions, *g*_*ij*_(*r*), derived from the MD–RMC models for *g*-Al_2_O_3_ and *l*-Al_2_O_3_ together with those for *g*-SiO_2_ are shown in Fig. 4b. *g*-SiO_2_ shows very prominent sharp Si–Si, Si–O, and O–O correlation peaks, whereas Al–Al, Al–O, and O–O correlation peaks are broader for *g*-Al_2_O_3_. Note that both the Al–O and O–O correlation peaks for *l*-Al_2_O_3_ are broader than those for *g*-Al_2_O_3_, whereas the Al–Al correlation peak in the former is identical to the latter. This trend is consistent with the finding that the difference in *S*^X^(*Q*) between *l*-Al_2_O_3_ and *g*-Al_2_O_3_ is very small.

Table 1 shows coordination number distributions and polyhedral connections in *g*-Al_2_O_3_, *l*-Al_2_O_3_, and *g*-SiO_2_. For a typical glass-forming oxide, *g*-SiO_2_, the number of oxygen atoms around a Si atom (*N*_A–X_) is 4, the number of Si atoms around an oxygen atom (*N*_X–A_) is 2, and the SiO_4_ polyhedra are connected via 100% corner-sharing, which is definitely in accordance with Zachariasen’s conventional glass formation concept. On the other hand, for *g*-Al_2_O_3_, more than 50% of the cations have *N*_A–X_ ≥ 5 and most of the oxygen atoms are connected with three Al atoms, showing the formation of OAl_3_ triclusters and a significant number of OAl_4_ tetraclusters. In addition, a significant fraction of edge-sharing AlO_*n*_ polyhedral units are observed in *g*-Al_2_O_3_. These features are completely inconsistent with Zachariasen’s rules. The fractions of AlO_5_ and AlO_6_ units, OAl_3_ triclusters and OAl_4_ tetraclusters, and edge-sharing AlO_*n*_ polyhedra are all characteristic features of a non-glass-forming behavior.

**Table 1:** Coordination number distributions and polyhedral connections in *g*-Al_2_O_3_, *l*-Al_2_O_3_, and *g*-SiO_2_.

	*N*_A–X_				*N*_X–A_				Polyhedral connection		
	3	4	5	6	2	3	4	5	Corner	Edge	Face
*g*-Al_2_O_3_	0.0	46.0	42.0	12.0	9.2	71.5	18.7	0.6	79.4	19.3	1.3
*l*-Al_2_O_3_	2.3	56.7	37.3	3.7	15.7	73.7	10.5	0.1	82.3	17.6	0.2
*g*-SiO_2_	0.1	99.8	0.1	0.0	99.9	0.1	0.0	0.0	100	0.0	0.0

**Figure 5:**
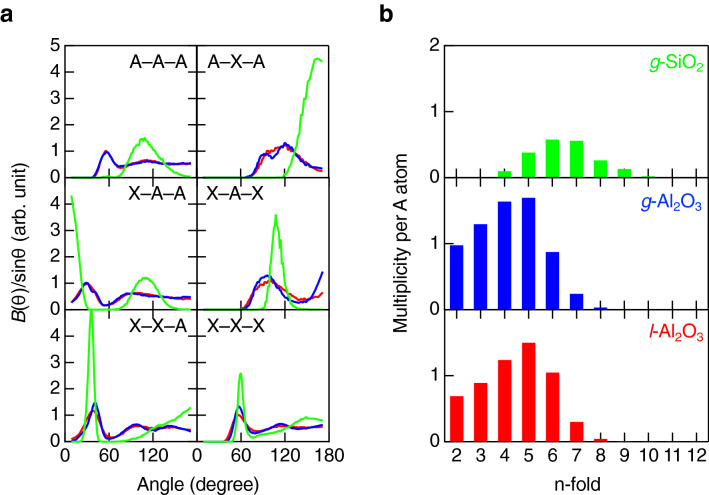
(**a**) Bond angle distributions for *g*-Al_2_O_3_, *l*-Al_2_O_3_, and *g*-SiO_2_. (**b**) Primitive ring size distributions for *g*-Al_2_O_3_, *l*-Al_2_O_3_, and *g*-SiO_2_. A = Si or Al and X = O.

To obtain the characteristic real space atomic arrangement of intermediate oxide glass, we analyzed the bond angle distribution. Figure 5a shows the bond angle distributions of *g*-Al_2_O_3_ and *l*-Al_2_O_3_ together with those of *g*-SiO_2_. The O–Si–O distribution has a well-defined peak at 109° attributable to the formation of regular SiO_4_ tetrahedra. The Si–Si–Si distribution has a broad peak at around 109°, suggesting the formation of SiSi_4_ hyper-tetrahedra^42^. The Si–O–Si distribution shows a peak at 165° attributable to the formation of the corner-sharing network. On the other hand, the bond angle distributions of *g*- and *l*-Al_2_O_3_ show completely different behaviors. The O–Al–O distributions of *g*-Al_2_O_3_ and *l*-Al_2_O_3_ shows peak at ~ 95 and ~ 180°, suggesting that AlO_*n*_ polyhedra are octahedral and are rather similar to those in non-glass-forming liquids, O–Zr–O in *l*-ZrO_2_^37^ and O–Er–O in *l*-Er_2_O_3_^41^. The Al–O–Al distributions has two peaks at 97 (edge-sharing) and 120° (OAl_3_ tricluster) of *g*-Al_2_O_3_, which become a broad single peak in *l*-Al_2_O_3_ owing to highly densely packed structure. Both the O–Al–O and Al–O–Al distributions of *g*-Al_2_O_3_ are slightly different from the results recently reported by Shi et al., because they do not have neutron diffraction data^15^. The most striking difference between *g*-SiO_2_ and *g*-/*l*-Al_2_O_3_ is the A–A–A distribution. The Si–Si–Si distribution suggests the formation of SiSi_4_ hyper-tetrahedra probably associated with the prominent FSDP, but the Al–Al–Al distribution shows two peaks at ~ 60° and ~ 115°, suggesting that the distribution of Al atoms is due to a typical dense random packing^43^, which cannot give rise to an FSDP in the diffraction data. The O–O–O distributions of *g*-Al_2_O_3_ and *l*-Al_2_O_3_ are also very different from that of *g*-SiO_2_ and suggest that the distributions of oxygen atoms are dense also due to the random packing. Moreover, it is suggested that the oxygen packing fraction of *g*-Al_2_O_3_ increases owing to the higher mass density in the glass in comparison with the liquid (see Table 1). Indeed, the distribution peak of *g*-Al_2_O_3_ is sharper than that of *l*-Al_2_O_3_. Both the O–O–Al and O–A–Al distributions of *g*-Al_2_O_3_ and *l*-Al_2_O_3_ are also different from the O–O–Si and O–S–Si distributions in *g*-SiO_2_.

To understand the topology of *g*-Al_2_O_3_ and *l*-Al_2_O_3_, we calculated the primitive ring size distribution and compared it with the *g*-SiO_2_ data in Fig. 5b. *g*-SiO_2_ shows a broad ring size distribution from threefold to ninefold rings, which is topologically disordered according to Gupta and Cooper^44^. Both *g*-Al_2_O_3_ and *l*-Al_2_O_3_ show broad ring size distributions that are nearly identical, but they have large fractions of small rings, e.g., twofold rings (edge-sharing) and threefold rings, which is a signature of the low glass forming ability of Al_2_O_3_.

**Figure 6:**
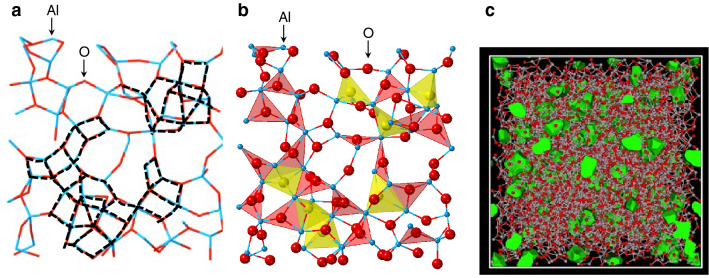
(**a**) Atomic configuration of *g*-Al_2_O_3_ (stick bonds schematic). (**b**) Atomic configuration of *g*-Al_2_O_3_ (schematic of the OAl_3_ triclusters (red) and OAl_4_ tetraclusters (yellow)). (**c**) Atomic configuration of *g*-Al_2_O_3_ with voids. Pink and red circles represent Al and O atoms, respectively, and green regions show the voids.

We show the atomic configuration of the glass obtained from the MD–RMC model in Fig. 6a to understand the structure of *g*-Al_2_O_3_. It is easily recognized that the assembly of two-membered rings (edge-sharing polyhedra) forms a lattice-like structure (black dotted line). The O–O atomic distance, which is the diagonal of one square, is ~ 2.3–2.7 Å, which is nearly consistent with the periodicity of ~ 2.3 Å estimated from the peak position of the PP observed in the *S*^N^(*Q*) for *g*-Al_2_O_3_. Therefore, we conclude that, in addition to the large fraction of corner-sharing OAl_3_ triclusters associated with the formation of octahedral AlO_*n*_ polyhedra, the larger fraction of edge-sharing AlO_*n*_ polyhedra for *g*-Al_2_O_3_ (19.3%) than for *l*-Al_2_O_3_ (17.6%) must be the origin of the exceptionally sharp PP observed in the *S*^N^(*Q*) for *g*-Al_2_O_3_. We show the OAl_3_ triclusters (red) and OAl_4_ tetraclusters (yellow) in Fig. 6b. Such a cluster network can be found in *g*-SiO_2_ at a high pressure of 200 GPa^45^, but it is possible to fabricate such a glass structure at ambient pressure through the electrochemical anodization process under high electric field^46,47^. The voids (highlighted in green) of *g*-Al_2_O_3_ are shown in Fig. 6c. The void volume ratio of *g*-SiO_2_ according to our previous study is 32%^5^_,_ whereas those in *g*-Al_2_O_3_ and *l*-Al_2_O_3_ is only 4.5% and 5.5%, respectively, indicating that a highly densely packed structure is formed in them. The dense-random-packing-like bond angle distribution (see Fig. 5a) with significantly octahedral AlO_*n*_ polyhedra is very different from that of conventional oxide glass but rather similar to that of metallic glass in which icosahedra is highly distorted owing to geometric frustration^48^.

The electrochemically prepared *g*-Al_2_O_3_ has many features that are completely outside of Zachariasen’s rules. Regardless of the dense oxygen packing structure with a large fraction of edge-sharing polyhedral motifs, *g*-Al_2_O_3_ can stably exist as glass. The electrochemical anodization technique can be regarded as a powerful tool for our questing for novel intermediate oxide glasses with an extremely dense structure, and the comprehensive understanding of the atomic structure of the glasses will give new insights into the fabrication of novel glass materials.
